# Methylation of *miR-155-3p* in mantle cell lymphoma and other non-Hodgkin's lymphomas

**DOI:** 10.18632/oncotarget.2390

**Published:** 2014-11-11

**Authors:** Rita Lh Yim, Kwan Yeung Wong, Yok Lam Kwong, Florence Loong, Chung Ying Leung, Raymond Chu, William Wai Lung Lam, Pak Kwan Hui, Raymond Lai, Chor Sang Chim

**Affiliations:** ^1^ Department of Medicine, Queen Mary Hospital, University of Hong Kong, Hong Kong; ^2^ Department of Pathology, Queen Mary Hospital, Hong Kong; ^3^ Department of Pathology, United Christian Hospital, Hong Kong; ^4^ Department of Pathology, Pamela Youde Nethersole Eastern Hospital, Hong Kong; ^5^ Department of Pathology, Princess Margaret Hospital, Hong Kong; ^6^ Department of Pathology, Kwong Wah Hospital, Hong Kong; ^7^ Department of Laboratory Medicine and Pathology, University of Alberta, Edmonton, Canada

**Keywords:** tumor suppressive microRNA, hypermethylation, miR-155-3p, mantle cell lymphoma, non-Hodgkin's lymphoma

## Abstract

Mantle cell lymphoma (MCL) is an aggressive B-cell non-Hodgkin's lymphoma (NHL). In cancers, tumor suppressive microRNAs may be silenced by DNA hypermethylation. By microRNA profiling of representative EBV-negative MCL cell lines before and after demethylation treatment, *miR-155-3p* was found significantly restored. Methylation-specific PCR, verified by pyrosequencing, showed complete methylation of *miR-155-3p* in one MCL cell line (REC-1). 5-aza-2′-deoxycytidine treatment of REC-1 led to demethylation and re-expression of *miR-155-3p*. Over-expression of *miR-155-3p* led to increased sub-G1 apoptotic cells and reduced cellular viability, demonstrating its tumor suppressive properties. By luciferase assay, *lymphotoxin-beta* (*LT-β*) was validated as a *miR-155-3p* target. In 31 primary MCL, *miR-155-3p* was found hypermethylated in 6(19%) cases. To test if methylation of *miR-155-3p* was MCL-specific, *miR-155-3p* methylation was tested in an additional 191 B-cell, T-cell and NK-cell NHLs, yielding *miR-155-3p* methylation in 66(34.6%) including 36(27%) non-MCL B-cell, 24(53%) T-cell and 6(46%) of NK-cell lymphoma. Moreover, in 72 primary NHL samples with RNA, *miR-155-3p* methylation correlated with *miR-155-3p* downregulation (p = 0.024), and *LT-β* upregulation (p = 0.043). Collectively, *miR-155-3p* is a potential tumor suppressive microRNA hypermethylated in MCL and other NHL subtypes. As *miR-155-3p* targets *LT-β*, which is an upstream activator of the non-canonical NF-kB signaling, *miR-155-3p* methylation is potentially important in lymphomagenesis.

## INTRODUCTION

Mantle cell lymphoma (MCL) is a rare but aggressive B-cell tumor accounting for 4%-6% of all non-Hodgkin's lymphomas (NHLs) [1-4]. It is distinguished from other NHLs by the genetic hallmark of t(11;14)(q13;32) translocation which leads to constitutive over-expression of cyclinD1 (*CCND1*) [1, 2, 5]. Other chromosomal losses reported in MCL include del(9p21), which encodes two tumor suppressive genes *p16^INK4a^* and *ARF* crucial for regulating cell cycle, in aggressive MCL cases [6, 7].

Unlike inactivating mutation of tumor suppressive genes, DNA methylation leads to reversible gene silencing mediated by the addition of a methyl group to the carbon 5 position of the cytosine ring in a CpG dinucleotide without changing of DNA sequence. Cancers are characterized by global DNA hypomethylation but locus-specific hypermethylation, particularly in promoter-associated CpG islands, leading to silencing of the downstream genes or non-protein-coding microRNAs [8-11]. Moreover, DNA methylation of genes or microRNAs may carry prognostic impacts in certain cancers including NHL [12, 13].

MicroRNAs are short, single-stranded, and non-protein-coding RNAs of 19-25 nucleotides [14]. Sequence-specific binding of a microRNA to 3′-untranslated region (3′-UTR) of its target protein-coding gene mRNA will result in translational block, and hence gene repression. We have previously reported methylation-silencing of several tumor suppressive microRNAs including *miR-129-2*, *miR-203* and *miR-124-1* in different types of haematological neoplasms, including B-, T- and NK-cell lymphoma [15-17]. However, data on the role DNA methylation-mediated silencing of tumor suppressive microRNA in MCL and other NHL subtypes remains scanty.

In this study, we attempted to identify tumor suppressive microRNAs potentially hypermethylated in MCL by studying microRNA expression profiles of representative EBV-negative MCL cell lines before and after treatment with 5-azadC, in which *miR-155-3p* emerged as an important candidate. Subsequent studies verified methylation of *miR-155-3p* in MCL cell lines and primary MCL samples, resulting in upregulation of *lymphotoxin-beta* (*LT-β*), which is a *miR-155-3p* direct target. To test if *miR-155-3p* methylation is MCL-specific, *miR-155-3p* methylation was studied in an additional 191 B- (other than MCL), T- and NK-cell lymphomas, which showed prevalent *miR-155-3p* methylation across multiple NHL subtypes. Moreover, in primary NHL samples with both DNA and RNA, *miR-155-3p* methylation correlated with repression of *miR-155-3p* and upregulation of *LT-β*. Since *LT-β* is a positive regulator of non-canonical NF-kB signaling, *miR-155-3p* methylation is implicated in lymphomagenesis.

## RESULTS

### Methylation-mediated silencing of *miR-155-3p* in MCL cell lines

**Figure 1:**
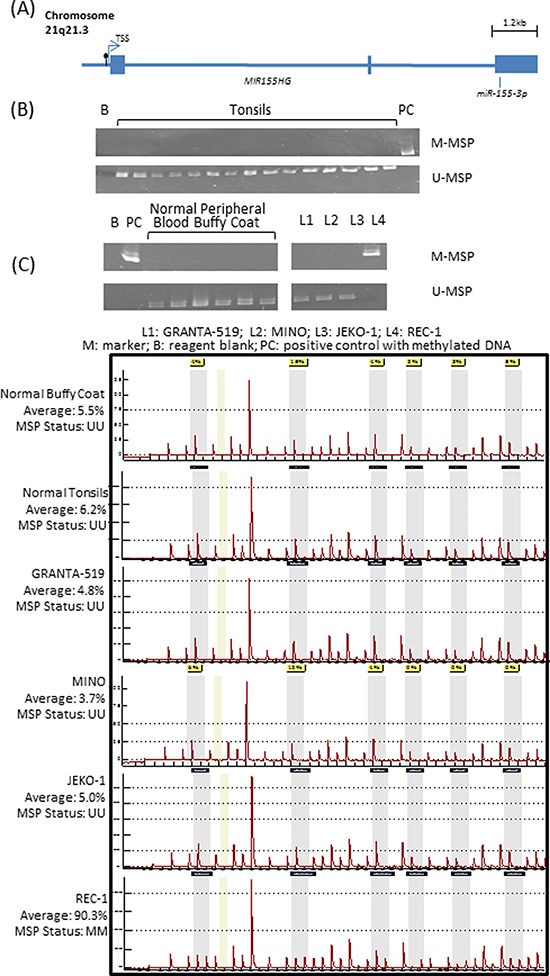
Methylation of *miR-155-3p* in mantle cell lymphoma (MCL) cell lines **(A)** Schematic diagram showing the exons (solid box) and CpG island (black lollipop) located 190bp upstream to the transcription start site (TSS) of *MIR155HG* which is the host gene for *miR-155-3p.* Primers for methylation-specific PCR (MSP) and quantitative pyrosequencing were designed to study that CpG island. **(B)** Methylation pattern in 6 normal buffy coats, 15 normal tonsils and 4 MCL cell lines by MSP detecting methylated [M] and unmethylated [U] allele separately. Complete methylation in REC-1 was detected by presence of M-MSP signal only. **(C)** Pyrograms of normal buffy coat, normal tonsil and 4 MCL cell lines measuring average methylation percentage of 6 consecutive CpG sites.

**Figure 2:**
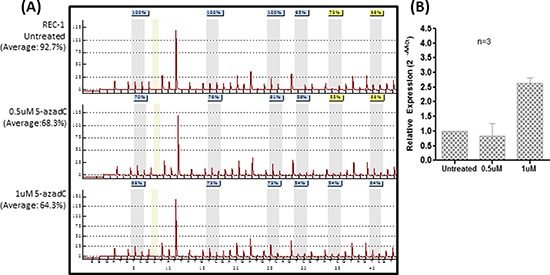
Tumor-specific methylation leads to silencing of *miR-155-3p* in REC-1 cells **(A)** Pyrograms of 5-azadC treated REC-1 cells for three days at concentrations of 0, 0.5 or 1uM. **(B)** RT-qPCR of REC-1 receiving 5-azadC treatment measuring expression of *miR-155-3p.*

MINO and JEKO-1 cells, before and after treatment with 1.5uM of 5-azadC, were subjected to high-throughput microarray detecting a total of the 754 known microRNAs. Upon 5-azadC treatment, expression of 26 and 44 microRNAs were found upregulated by ≥ 2.5-fold in MINO and JEKO-1 respectively (Supplementary Table 1). One of the most upregulated microRNAs was *miR-155-3p*, which showed a 33-fold upregulation in MINO. By studying methylation status of the promoter region of its host gene, *MIR155HG* (Figure 1A), methylation was absent in healthy buffy coat and tonsil controls by MSP (Figure 1B). By MSP, complete methylation was detected in REC-1, one of the four MCL cell lines. Moreover, the methylation status detected by MSP was verified by quantitative pyrosequencing, which showed an average methylation frequency of <10% in completely unmethylated normal controls (buffy coat from healthy blood donor and normal tonsils) and completely unmethylated cell lines. By contrast, an average methylation frequency of >90% was detected in REC-1 which was completely methylated by MSP (Figure 1C). Since REC-1 was completely methylated, this cell line was treated with different concentrations of 5-azadC for 3 days to investigate if demethylation could restore expression of *miR-155-3p.* Treatment with 5-azadC resulted in a dose-dependent demethylation as measured by pyrosequencing (Figure 2A) and emergence of U-MSP amplification by MSP (data not shown). Importantly, *miR-155-3p* expression was restored accordingly upon treatment indicating the reversible gene silencing of this microRNA (Figure 2B).

### Tumor suppressive function of *miR-155-3p* in MCL

**Figure 3:**
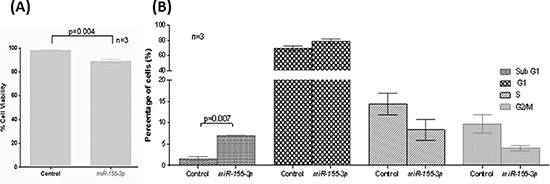
Tumor suppressive function of *miR-155-3p* REC-1 cells overexpressed with *miR-155-3p* for 24hours **(A)** Trypan blue staining to measure cell viability and **(B)** 7-AAD staining for cell cycle analysis.

To study the biological function of *miR-155-3p* in MCL, *miR-155-3p* was over-expressed in REC-1 followed by trypan blue exclusion assay (Figure 3A) and cell cycle analysis (Figure 3B). Restoration of *miR-155-3p* expression led to decreased number of viable cells by 10% (p = 0.004) as detected by trypan blue staining and increased Sub-G1 apoptotic cells by 4.4-fold (p = 0.007) as demonstrated by cell cycle analysis compared with cells transfected with non-targeting scramble control. Therefore, *miR-155-3p* might function as a tumor suppressive microRNA in MCL.

### LT-β targeted by *miR-155-3p*

**Figure 4:**
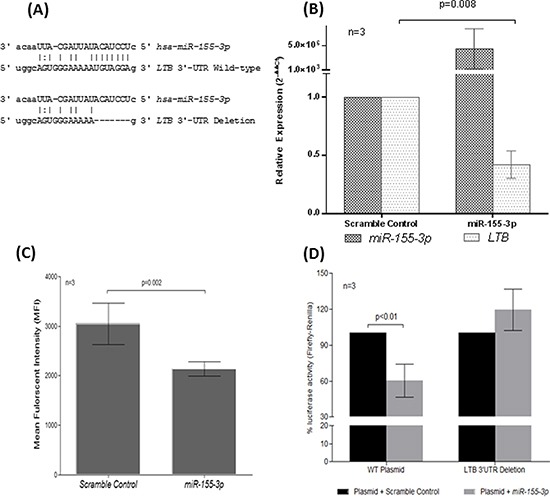
Direct regulation of LT-β by *miR-155-3p* through complementarity to 3′-UTR of *LT-β* **(A)** Sequence alignment of *miR-155-3p* seed sequences with the binding region of the *LT-β* 3′-UTR. The 3′-UTR sequence of the wild-type (LTB 3′-UTR Wild-type) or the 7bp-deletion mutant (LTB 3′-UTR Deletion) cloned into luciferase plasmid was depicted. Expression analysis of LT-β in REC-1 cells overexpressed with *miR-155-3p* by **(B)** RT-qPCR and **(C)** flow cytometry. **(D)** Luciferase reporter assay using luciferase plasmid carrying wild-type (WT) or deleted binding site (LTB-Deleted) of *LT-β* 3′UTR co-transfected with *miR-155-3p* as compared with scramble nucleotide control in HeLa cells.

To identify protein-coding genes (PCGs) targeted by *miR-155-3p*, bioinformatic analysis of the 3′-UTR of PCGs using 3 databases (miR-DB, RNA22 and miRanda) was performed. In brief, the putative target fulfilled the following criteria: (1) it was detected as putative target in all three databases, (2) it has been reported to play a role in B-cell development and was found overexpressed in MCL, other NHL subtypes or other cancers, (3) it was a positive regulator that led to activation of signaling pathways that were activated in MCL (e.g. NF-kB, ERK and AKT) or in other cancers, and (4) the target had a typical 7-8mer (8mer, 7mer-m8, 7mer-A1) match to the *miR-155-3p* seed region as predicted by the databases. *LT-β* appeared in all 3 databases as one of the best targets for the *miR-155-3p*. By studying the sequence alignment, the 3′-UTR of *LT-β* contains 1 sequence motif that perfectly matched with the seed region of the *miR-155-3p* (i.e. nts 2-9 at the 5′-end of the mature microRNA) (Figure 4A). Restoration of *miR-155-3p* in REC-1 led to downregulation of LT-β expression at both RNA (by RT-qPCR; Figure 4B) and protein level (by flow cytometry; Figure 4C), suggesting that LT-β expression was repressed by *miR-155-3p*.

Therefore, luciferase reporter assay was performed to validate if *LT-β* is a direct target of *miR-155-3p.* Co-transfection of a luciferase reporter construct, containing a wild-type 3′-UTR sequence of the *LT-β*, with *miR-155-3p* resulted in loss of luciferase activity by 40% as compared with non-targeting scramble oligonucleotides (Figure 4D). Conversely, the luciferase activity was fully rescued when a mutant luciferase reporter, which carried a deletion of the seed region binding site of *miR-155-3p*, was co-transfected with *miR-155-3p*. These findings collectively suggested the direct binding and inhibition of the *LT-β* 3′-UTR by *miR-155-3p*.

### Methylation-mediated silencing of *miR-155-3p* in MCL and other NHL patients

**Figure 5:**
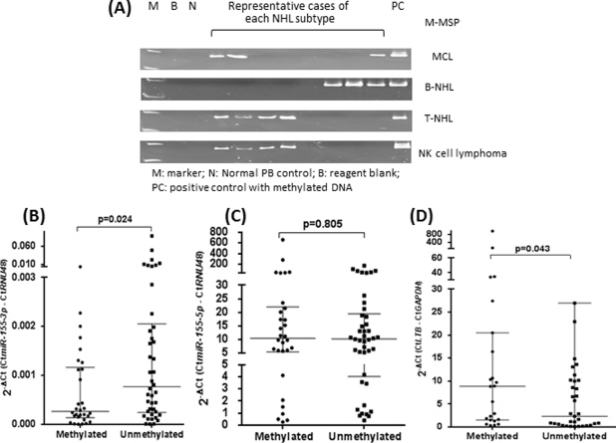
Overexpression of *LT-β* resulted from methylation-mediated silencing of *miR-155-3p* **(A)** Representative cases showing methylation of *miR-155-3p* in 31 MCL and 133 non-MCL B-NHL, 45 T-NHL and 13 NK cell lymphoma biopsies using M-MSP. **(B)** Expression analysis of *miR-155-3p* a total of 72 NHL patient biopsies with or without methylations as detected by M-MSP. **(C)** Expression analysis of *miR-155-5p* the same 72 NHL patient biopsies with or without methylation as detected by M-MSP. **(D)** Expression analysis of *LT-β* in a random cohort (n = 34) of NHL patient biopsies with or without *miR-155-3p* methylation.

In the 31 primary MCL samples, aberrant methylation was also found in 6(19.4%) cases, suggesting a pathological relevance of methylation-mediated silencing of *miR-155-3p* (Figure 5A). To study if *miR-155-3p* methylation was restricted to the MCL, which carried the characteristic t(11;14)(q13;32) translocation, *miR-155-3p* methylation was also studied in a panel of NHLs other than MCL, which comprised 133 non-MCL B-cell, 45 T-cell and 13 NK-cell neoplasms. *miR-155-3p* was found to be methylated in 36(27.0%) of non-MCL B-NHL, 24(53.3%) of T-NHL and 6(46.2%) of NK-cell lymphoma (Table 2), being more frequent in NK or T-NHL than B-NHL (p = 0.003). In 72 NHL patients with frozen tissues, *miR-155-3p* expression correlated inversely with methylation status, which was demonstrated by lower *miR-155-3p* expression in patients with *miR-155-3p* methylation than those without (Figure 5B) (p = 0.024). Since both *miR-155-3p* and *miR-155-5p* may be generated from the same precursor, expression of *miR-155-5p* was also studied in primary lymphoma samples in which both DNA & RNA were available. In contrast to *miR-155-3p*, there was no correlation between methylation status and expression of *miR-155-5p* (Figure 5C) (p = 0.805). Therefore, DNA methylation was likely more important in regulating *miR-155-3p* than *miR-155-5p*.

### Overexpression of *LT-β* mediated by *miR-155-3p* methylation in primary NHL samples

To validate if methylation-mediated silencing of *miR-155-3p* could lead to overexpression of *LT-β* in primary MCL and other NHLs, the expression of *LT-β* was profiled in 34 patients with frozen specimens (Figure 5D). Indeed, specimens methylated for *miR-155-3p* showed higher *LT-β* expression compared with unmethylated specimens (p = 0.043).

## DISCUSSION

MCL is a rare entity, and comprised only 4% of all NHL in Hong Kong [3], hence only 31 cases were available for study here. Nonetheless, it is one of the largest analyses of microRNA methylation in MCL.

In this study, we have shown that *miR-155-3p* might be a novel tumor suppressive microRNA silenced in MCL due to the aberrant DNA methylation. However, *miR-155-3p* methylation is not restricted to MCL, and occurs in a wide array of other B-, T- and NK-cell lymphoma, hence suggesting a potential role in lymphomagenesis. This is consistent with our previous studies of *miR-203, miR-124-1, miR-129-2* and *miR-34a* in NHLs, in which methylation of these tumor suppressive microRNAs occurred in multiple subtypes of NHLs [15-18]. Therefore, it is unlikely that methylation of *miR-155-3p* and these microRNAs are governed by the primary genetic translocation involved in the pathogenesis of disease. Other microRNAs downregulated by promoter DNA methylation includes *miR-29a* and *miR-146a* [19, 20]. In anaplastic lymphoma kinase expressing ALCL (ALK+ve ALCL) with t(2;5) translocation, *miR-29a* has been shown to target, and hence downregulate an anti-apoptotic protein, MCL-1 [19]. Similarly, *miR-146a* has been shown hypermethylated in NK/T-cell lymphoma, in which *miR-146a* was shown to target TRAF6 and hence downregulate NF-kB signaling [20].

Secondly, we have demonstrated *miR-155-3p* might function as a tumor suppressi*v*e microRNA in MCL cells by regulating the expression of LT-β. Indeed, the LT-β/LTβR axis has been shown to be important in physiological development of secondary lymphoid tissues. Interaction of LTα_1_β_2_ heterotrimer on immune cells with LTβR on stromal cell results in activation of non-canonical NF-kB signaling in stromal cells, with upregulation of adhesion molecules and secretion of chemokines for B- and T-cells, thereby attracting and retaining additional immune cells for the growth of lymph nodes and Peyer's patches [21, 22]. Consistent with this notion, genetic knockout of *LT-β* or *LTβR* gene in mice has been shown to result in abnormal development or complete loss of lymph nodes and Peyer's patches [23, 24]. On the other hand, an oncogenic role of LT-β/LTβR axis has been demonstrated in a mouse model, in which disease progression was associated tumor infiltration of LT-β-expressing B-cells, which interacted with LTβR-expressing tumor cells led to induction of IKK-α nuclear translocation and NF-kB activation [25]. Moreover, both ligand (LT-β) and receptor (LTβR) have been shown expressed in non-Hodgkin's lymphoma cells including MCL [26], which potentially leads to constitutive activation of NF-kB signaling [27], suggesting a pathogenic role of the LT-β/LTβR axis in lymphomagenesis [28-30]. Using transgenic mouse model which displayed an aggressive B-cell lymphoma phenotype, LTβR signalling has been shown to collaborate with CCR7 signaling to maintain T-cell and B-cell localization within the lymphoid microenvironment hence contribution to lymphomagenesis. Inhibition of LTβR signaling by anti- LTβR antibody or knockdown of LTα impaired cell interaction between lymphoma cell and stromal cell, potentially by disrupting production of CCL19 and CCL21 by stromal cells which are ligands for CCR7 [31]. Furthermore, in this study, in primary lymphoma samples with frozen tissue, which included MCL and other lymphoma subtypes, *LT-β* expression correlated inversely with *miR-155-3p* methylation, hence consistent with LT-β as a direct target of *miR-155-3p* in multiple lymphoma subtypes in addition to MCL. Taken together, we have demonstrated a role of *miR-155-3p* methylation in the regulation of LT-β/LTβR axis in MCL and other NHL subtypes. Therefore, whether *miR-155-3p* methylation leads to dysregulation of NF-kB signaling in lymphomagenesis warrants further study.

In general, biogenesis of microRNA results in generation of two mature microRNAs, which are denoted as 5p and 3p, with different sequences from a same primary transcript [32]. Despite that majority of cancer studies focused on 5p microRNAs, recent studies demonstrated tumor suppressor property associated with several 3p microRNAs including *miR-574-3p*, *miR-219-2-3p* and *miR-338-3p* in various solid cancers [33-37]. For instance, under-expression of *miR-574-3p* correlated with advanced pathological stage in prostate cancer. In cell lines, restoration of *miR-574-3p in vitro* induced apoptosis. Moreover, mice injected with prostate cancer cells overexpressed with *miR-574-3p* had retarded tumor growth compared to mice injected with untransfected tumor cells, testifying the tumor suppressive role of *miR-574-3p* [36]. Similarly, *miR-219-2-3p* has been shown to induce apoptosis, inhibit cell proliferation and metastasis in gastric cancer *in vitro*, and hence a *bona fide* tumor suppressor microRNA [34]. Moreover, in primary gastric cancer samples, downregulation of *miR-219-2-3p* associated with DNA methylation, also correlated with advanced tumor stage and grade. In addition, tumor suppressive function of *miR-338-3p*, downregulated in neuroblastoma, gastric and colorectal cancer, has been demonstrated in these tumors [33, 35, 37, 38]. Similar to *miR-219-2-3p*, *miR-338-3p* has been shown to be hypermethylated in gastric cancer, and restored *miR-338-3p* expression induces apoptosis *in vitro* and inhibits tumor development *in vivo* [39]. Finally, *miR-338-3p* targets PREX2a, which plays an oncogenic role in activating AKT signaling in gastric cancer and neuroblastoma [33, 37]. Collectively, these studies illustrate the tumor suppressive function of 3p microRNAs through regulation of cell survival and signaling pathway.

In conclusion, we have identified *miR-155-3p* might be a novel tumor suppressive microRNA silenced in MCL due to promoter hypermethylation of its host gene *MIR155HG*, resulting in upregulation of LT-β. In addition to MCL, *miR-155-3p* methylation is prevalent in other B-, T- and NK-cell NHLs, which correlated with over-expression LT-β, and hence potentially important in pathogenesis of lymphoma.

## MATERIALS AND METHODS

### Patient Samples

Thirty-one primary MCL samples were obtained from the Department of Pathology from Queen Mary Hospital since 1997. Diagnosis of MCL was based on morphologic criteria. Immunophenotyping was performed on cryostat sections or paraffin sections with standard immunoperoxidase technique. Paraffin sections of formalin- or B5-fixed tissue were stained with hematoxylin-eosin to confirm the diagnosis of lymphoma and examined for the expression of B- and T-cell markers. The panel of antibodies used included CD3 (Leu4; Becton Dickinson, San Jose, CA), CD3 (polyclonal; Dako, Glostrup, Denmark), CD5 (Dako), CD10 (J5; Coulter, Hialeah, FL), CD19 (Leu 12; Becton Dickinson), CD20 (L26; Dako), CD22 (Dako), CD23 (Dako), and cyclin D1 (Zymed, San Francisco, CA). All 31 cases of MCL were classic-variant, with the phenotype of CD5+, CD10−, and CD23−, and immunoreactive for cyclin D1[40]. Moreover, an additional 191 diagnostic lymph node biopsies were obtained from five major hospitals (Queen Mary Hospital, Pamela Youde Nethersole Eastern Hospital, United Christian Hospital, Kwong Wah Hospital and Princess Margaret Hospital) including 133 B-NHL other than MCL, 45 T-NHL and 13 NK-cell lymphomas. Diagnoses of NHLs were made according to WHO (World Health Organization) classification [41]. Among the non-MCL B-NHL cases, 57 each were diffuse large B-cell lymphoma (DLBCL) and follicular lymphoma (FL) respectively, 6 cases were Burkitt-like lymphoma (BL), 5 cases were mucosa-associated lymphoid tissue lymphoma (MALT), and 8 were marginal zone B-cell lymphoma (MZBCL). Of the 45 T-NHL cases, 21 were classified as peripheral T-cell lymphoma (PTCL) while 24 were classified as angioimmunoblastic T-cell lymphoma (AITL). Formalin-fixed, paraffin-embedded (FFPE) tonsils from 15 healthy individuals undergoing tonsillectomy were also collected. Moreover, frozen tissues, in which both DNA and RNA were extracted, were available in 72 patients including 4 MCLs, 5 BLs, 34 DLBCLs, 8 FLs, 7 MZBCLs and 14 AITLs. Informed consents were obtained from all patients and the study was approved by Institutional Review Board of Queen Mary Hospital.

### Plasmid Constructs

The *LT-β* 3′-UTR cDNA sequence (NM_002341.1 or NM_009588.1) of a total of 157bp flanking the seed region binding site of mature *miR-155-3p* was cloned into SacI and XbaI restriction sites of pmirGLO luciferase reporter (Promega, Madison, WI, USA), encoding both firefly luciferase for reporter signal and renilla luciferase for assay normalization. A *LT-β* 3′-UTR mutant control construct, in which the sequence complementary to the seed region binding site of *miR-155-3p* was deleted, was generated using QuikChange XL Site-Directed Mutagenesis Kit (Stratagene, La Jolla, CA, USA) following manufacturer's instruction. Primer sequences used were listed in Table 1.

**Table 1:** Primer sequences for Methylation-specific PCR (MSP), Pyrosequencing, Real-time PCR and luciferase reporter assay

Forward Primer		Reverse Primer	Sequencing Primer
**Methylation-specific PCR (MSP)**			
**M-MSP**	TAG TCG ATT GAA AGT TCG GGC	CCT TTC TCG TAA ATC ATT ACG	
**U-MSP**	TTT TAG TTG ATT GAA AGT TTG GGT	TTT CCC TTT CTC ATA AAT CAT TAC A	
**Quantitative Bisulfite Pyrosequencing**			
	GGT TTT TTG TAA GGA GAG AGT AGA GAT	AAT ATT TTC CCC TTT CCC TTT CT	ATA TTT TTG TTA TTT AGT TGT AAG A
**Real-time PCR**			
*LT-β*	GGT TTC AGA AGC TGC CAG AG	TTC AGC GGA GCG CCT AT	
*GAPDH*	ACC ACA GTC CAT GCC ATC ACT	TCC ACC ACC CTG TTG CTG TA	
**Luciferase Reporter Plasmid**			
*LT-β* **Wild-type**	CGA GCT CGG GGA ATA TGA GTG CGT GGT G	GCT CTA GAG CTC TAG ATT TAT CGG CAG CAC TGA AGC	
*LT-β* **Deletion**	CCC ATG GCA GTG GGA AAA AGA CTG TTT GGA AAT TG	CAA TTT CCA AAC AGT CTT TTT CCC ACT GCC ATG GG	

### Cell Culture

Four MCL cell lines (REC-1, GRANTA-519, MINO & JEKO-1) were used in this study. REC-1 was kindly provided by Prof Raymond Lai (Department of Laboratory Medicine and Pathology, University of Alberta and Cross Cancer Institute). Other cell lines were purchased from Deutsche Sammlung von Mikroogranismen und Zellkulturen (DSMZ) (Braunschweig, Germany). All cell lines were maintained in RPMI-1640 supplemented with 15% fetal bovine serum, 50U/mL of penicillin and 50ug/mL streptomycin in a humidified atmosphere of 5% CO_2_ at 37^o^C.

### Hypomethylating Treatment

JEKO-1, MINO and REC-1 were seeded at a density of 1×10^6^ cells/ml and cultured with 0.5–1.5uM of 5-aza-2′-deoxycytidine (5-azadC) (Sigma–Aldrich) for 3-5 days as previously described [15, 18, 42].

### DNA and RNA Extraction

DNA isolation from frozen patient biopsies was performed by automated DNA extraction system (DNA Tissue Kit from Qiagen) while FFPE fixed NHL samples and the 15 normal tonsils were extracted with QIAamp DNA FFPE Tissue Kit (Qiagen). For cultured cell lines, DNA extraction was performed using DNA Blood Mini (Qiagen). Extractions of microRNA were performed by using mirVanaTM miRNA Isolation Kit (Ambion) following manufacturer's instruction.

### Microarray Analysis

Total RNA isolated from MINO and JEKO-1 before and after 5-azadC treatment were converted into cDNA by Megaplex^TM^ RT Primers and TaqMan® MicroRNA Reverse Transcription Kit. cDNA was pre-amplified using Megaplex^TM^ PreAmp Primer and loaded onto 384-well format Taqman® human microRNA array A V2.0 & B V3.0. Real-time PCR was performed on 7900HT Real-Time PCR system and raw data were analyzed normalizing to mean of three endogenous controls (*U6snRNA*, *RNU44* and *RNU48*). Relative microRNA levels were determined by ΔΔCt using endogenous controls and untreated controls using SDS 2.4 and RQ manager 1.2. All experimental procedures and analyses were performed according to manufacturer's instruction, using reagents, system and softwares acquired from Applied Biosystems (Foster City, USA). Candidate microRNAs were selected according to the following criteria: (1) upregulated by ≥ 2.5-fold in either or both cell lines after 5-azadC treatment, (2) localized outside the X chromosome or imprinted regions (e.g. 14q32) due to potential methylation in normal tissues, (3) presence of CpG island, (4) absence of methylation in healthy controls, and (5) showed complete methylation in any of the MCL cell lines.

**Table 2:** Frequency of *miR-155-3p* methylation in MCL and different types of NHLs as detected by MSP

Disease Subtype	Sample Size	Frequency Methylated (%)
**Mantle cell lymphoma (MCL)**	31	6 (19.4%)
**Other B-NHL**	133	36 (27.0%)
**Diffuse large B-cell lymphoma (DLBCL)**	57	18 (31.5%)
**Follicular lymphoma (FL)**	57	11 (19.3%)
**Burkitt -like (BL)lymphoma**	6	2 (33.3%)
**Mucosa-associated lymphoid tissue lymphoma (MALT)**	5	2 (40.0%)
**Marginal Zone B-cell lymphoma (MZBCL)**	8	3 (37.5%)
**T-NHLs**	45	24 (53.3%)
**Peripheral T-cell Lymphoma (PTCL)**	21	11 (52.4%)
**Anaplastic T-cell lymphoma (AITL)**	24	13 (54.2%)
**NK cell lymphoma**	13	6 (46.2%)

### Methylation-specific Polymerase Chain Reaction (MSP)

DNA samples were treated to convert unmethylated cytosine into uracil by EpiTect Bisulfite Kit (Qiagen). As *miR-155-3p* is an exonic microRNA localized to the third exon of and regulated by the same promoter of its host gene, *MIR155HG* (previously known as *B-cell integration cluster*, *BIC*), primers used for methylated-MSP (M-MSP) and unmethylated-MSP (U-MSP) were designed at CpG island upstream of its host gene of *MIR155HG* (Table 1) [43, 44]. All MSPs were conducted as previously described [15, 18, 42]. DNA from 6 normal peripheral blood and 15 normal tonsils were used as unmethylated controls while an enzymatically methylated control DNA purchased from CpGenome Universal Methylated DNA (Chemicon) was used as methylated control.

### Quantitative Bisulfite Pyrosequencing

Using bisulfite converted DNA as template, a segment of promoter region overlapping with the amplicon of the MSP was amplified with a primer set, which amplified both methylated and unmethylated DNA sequences in an unbiased manner. Subsequently, a stretch of DNA containing 6 consecutive CpG dinucleotides on the PCR products was pyrosequenced and quantified as previously described [17]. Quantitative DNA methylation analysis was carried out on a PSQ 96MA system and results were analyzed using PyroQ-CpG 1.0.9. Primer sequences for PCR amplification and pyrosequencing were designed using PyroMark Assay Design 2 (Table 1).

### RT-qPCR

For expression analysis of mature *miR-155-3p* or *miR-155-5p*, RNA was reverse transcribed into cDNA by TaqMan® MicroRNA Reverse Transcription Kit, followed by RT-qPCR using TaqMan® MicroRNA Assays (Applied Biosystems). *RNU48* was used as endogenous control as previously described [15, 18, 42]. Conventional RT-qPCR was employed for the analysis of *LT-β*, using QuantiTect Reverse Transcription Kit (Qiagen) and iQTM SYBR® Green Supermix (Bio-Rad) according to manufacturer's instructions. *GAPDH* was included as endogenous control. For each sample, signals from triplicate wells were recorded and calculated as mean of Ct for analysis. All data were analyzed adopting the 2^−ΔΔCt^ or 2^−ΔCt^ method and primer sequences for *LT-β* and *GAPDH* were listed in Table 1.

### *miR-155-3p* Overexpression

Exponentially growing cells at a density of 1×10^6^ cells/ml were transfected with 50nM of either *miR-155-3p* mimic oligonucleotide or a scramble negative control (Ambion), using Lipofectamine 2000 transfection reagent (Invitrogen) according to manufacturer's instruction.

### Trypan Blue Exclusion Assay

Viable live cells were distinguished by the exclusion of trypan blue staining as observed under microscope. Five random microscopic fields were counted and percentage of viable cells was calculated as

$$\frac{\mathrm{total number of live cells per microscopic field}}{\mathrm{total number of cells per microscopic field}}\times 100\%$$

### Cell Cycle Analysis

Harvested cells were fixed and permeabilized with Cytofix/Cytoperm buffer (BD Biosciences) followed by 80% ethanol at −20°C for 2 hours. Cells were then washed and stained with 7-AAD (BD Bio sciences) according to manufacturer's instruction. Fluorescence readings were acquired using a BD LSR Fortessa analyser (BD Biosciences). Quantitation of singlets at G1, S, G2 and M phases in addition to Sub-G1 fractions was performed using FlowJo 7.6.1 (Tree Star, Inc).

### Flow Cytometric Staining

Harvested cells of 1×10^6^ were washed with PBS followed by fixation and permeabilization using Cytofix/Cytoperm buffer (BD Biosciences) according to manufacturer's instruction. Cells were then stained with anti-LT-β rabbit polyclonal antibody at a final concentration of 4ng/mL (Abcam, Cat# ab64835) followed by secondary incubation with Alexa Fluor 594 goat anti-rabbit IgG at a concentration of 10ng/mL (Molecular Probe, Cat# A11072), as recommended by manufacturer.

### Luciferase Reporter Assay

Wild-type or mutant *LT-β* 3′-UTR luciferase reporter construct was co-transfected with *miR-155-3p* oligo mimic or scramble negative control (Ambion) into HeLa cells (kindly provided by Dr Zou, at Department of Medicine, The University of Hong Kong) using Lipofectamine 2000 (Invitrogen) [45]. After 24h, the luminescent signal produced by Dual-Glo Luciferase Assay system (Promega) was measured using FLUOstar OPTIMA (BMG Labtech), following the manufacturer's instructions. A triplicate reading was recorded for each exogenous transfection.

### Statistical Analysis

Mean expression of microRNAs or genes in methylated and unmethylated NHL cases were compared by the Student's t-test. Frequency of *miR-155-3p* methylation in different NHL subtypes was compared by X^2^ test. The means of three independent 5-azadC treatments or exogenous transfections were compared by Student's t-test. All p-values were 2-sided and statistical significance was defined as p-value < 0.05.

## SUPPLEMENTARY TABLE



## Abbreviations

3′-UTR: 3′-untranslated region
5-azadC: 5-aza-2′-deoxycytidine
AITL: angioimmunoblastic T-cell lymphoma
BL: Burkitt-like lymphoma
CCND1: cyclin D1
DLBCL: diffuse large B-cell lymphoma
DSMZ: Deutsche Sammlung von Mikroogranismen und Zellkulturen
FFPE: formalin-fixed, paraffin-embedded
FL: follicular lymphoma
LT-β: lymphotoxin-beta
MCL: Mantle cell lymphoma
MSP: methylation-specific PCR
M-MSP: methylated-MSP
MZBCL: marginal zone B-cell lymphoma
NHL: non-Hodgkin's lymphoma
NK: natural killer
PCGs: protein-coding genes
PTCL: peripheral T-cell lymphoma
U-MSP: unmethylated-MSP
WHO: World Health Organization
