# Supplementary material for: Methylation of miR-155-3p in mantle cell lymphoma and other non-Hodgkin's lymphomas
Source: Oncotarget. 2014 Nov 11;5(20):9770–82. doi: 10.18632/oncotarget.2390 (PMC4259436; doi:10.18632/oncotarget.2390)
Supplement: Supplementary file 1 [file oncotarget-05-9770-s001.pdf]

SUPPLEMENTARY TABLE

Supplementary Table S1. List of microRNAs upregulated by ≥2.5-fold in either MINO or JEKO-1 upon 5-azadC treatment

**MINO**	
**microRNA**	**Fold-change**
hsa-let-7a-000377	5.3673
hsa-let-7f-000382	22.5855
hsa-miR-1271-002779	4.1095
hsa-miR-146b-001097	2.6759
hsa-miR-155#-002287	32.9479
hsa-miR-18b-002217	2.6408
hsa-miR-200b-002251	21.8606
hsa-miR-200c-002300	5.4204
hsa-miR-212-000515	11.1512
hsa-miR-24-2#-002441	33.759
hsa-miR-29b-000413	22.4087
hsa-miR-30a-5p-000417	15.9474
hsa-miR-328-000543	21.6568
hsa-miR-338-5P-002658	14.072
hsa-miR-345-002186	2.7101
hsa-miR-34a-000426	21.8999
hsa-miR-374-000563	2.6872
hsa-miR-381-000571	2.6494
hsa-miR-423-5p-002340	21.6898
hsa-miR-501-001047	11.1529
hsa-miR-502-3p-002083	22.2829
hsa-miR-550-001544	33.1601
hsa-miR-664-002897	132.9803
hsa-miR-708-002341	22.1625
hsa-miR-874-002268	21.8255
hsa-miR-92a-1#-002137	33.8066
**JEKO-1**	
**microRNA**	**Fold-change**
hsa-let-7c-000379	36.0442
hsa-let-7d-002283	5.1039
hsa-miR-101-002253	100.0538
hsa-miR-1208-002880	32.0268
hsa-miR-1225-3P-002766	31.6219
hsa-miR-1276-002843	15.9905

microRNA	Fold-change
hsa-miR-128a-002216	4.961
hsa-miR-1290-002863	7.885
hsa-miR-130b-000456	2.519
hsa-miR-132-000457	80.6124
hsa-miR-142-5p-002248	4.9335
hsa-miR-152-000475	160.8948
hsa-miR-15a#-002419	14.7313
hsa-miR-181a-000480	5.1392
hsa-miR-181a-2#-002317	3.8954
hsa-miR-18b-002217	2.557
hsa-miR-21-000397	4.8801
hsa-miR-27a#-002445	3.9299
hsa-miR-27a-000408	4.8691
hsa-miR-27b#-002174	62.9708
hsa-miR-29a-002112	5.0043
hsa-miR-29c-000587	4.9219
hsa-miR-323-3p-002227	39.2982
hsa-miR-328-000543	81.0661
hsa-miR-337-5p-002156	40.1739
hsa-miR-338-5P-002658	15.4996
hsa-miR-340-002258	9.8117
hsa-miR-34a-000426	39.6942
hsa-miR-361-3p-002116	62.8983
hsa-miR-381-000571	9.8025
hsa-miR-422a-002297	4.7736
hsa-miR-487a-001279	82.0009
hsa-miR-532-001518	5.0264
hsa-miR-580-001621	63.6942
hsa-miR-582-3p-002399	37.6819
hsa-miR-590-5p-001984	79.8172
hsa-miR-641-001585	31.4593
hsa-miR-664-002897	3.845
hsa-miR-672-002327	82.6162
hsa-miR-708-002341	4.9601
hsa-miR-769-5p-001998	62.4961
hsa-miR-885-5p-002296	80.1506
hsa-miR-886-5p-002193	4.997
hsa-miR-9#-002231	63.4836
